# DNA methylation mediated down-regulation of ANGPTL4 promotes colorectal cancer metastasis by activating the ERK pathway

**DOI:** 10.7150/jca.52338

**Published:** 2021-07-13

**Authors:** Kunning Zhang, Zhiwei Zhai, Sanshui Yu, Yu Tao

**Affiliations:** 1 Department of Pathology, Beijing Chao-Yang Hospital, Capital Medical University, Beijing 100020, P.R. China; 2 Department of General Surgery, Beijing Chao-Yang Hospital, Capital Medical University, Beijing 100020, P.R. China

**Keywords:** Colorectal cancer, DNA methylation, Angiopoietin-like 4, Carcinoma-associated fibroblasts, Epithelial-mesenchymal transition

## Abstract

**Background:** Colorectal cancer (CRC) imposes significant health burden and is increasing in incidence. NGPTL4 has been implicated in the development of CRC. The present study aimed to investigate the molecular mechanisms by which ANGPTL4 expression might regulate epithelial-mesenchymal transition (EMT) and the tumor microenvironment in CRC.

**Methods:** CRC and para-carcinoma tissues were collected from 67 CRC patients. ANGPTL4 expression levels and DNA methylation of ANGPTL4 promoter region were determined. Next, the migration and invasion capacities of CRC cells were assessed. Immunofluorescence and Western blot were used to identify the signaling pathways by which ANGPTL4 mediated tumor metastasis. A tumorigenesis mice model with transplanted fibroblast cells and ANGPTL4 overexpressed CRC cells was established to investigate the effects of ANGPTL4 on the metastasis of cancer cells *in vivo*.

**Results:** ANGPTL4 was significantly decreased in CRC tissues and DNA hypermethylation was involved in the regulation of ANGPTL4. Mechanistically, ANGPTL4 induced activation of cancer-associated fibroblasts in the tumor microenvironment and promoted EMT in CRC cells through the ERK signaling pathway. *In vivo*, the overexpression of ANGPTL4 was found to inhibit the metastasis of tumor cells in lung tissues.

**Conclusion:** DNA hypermethylation induced ANGPTL4 downregulation promoted the activation of cancer-associated fibroblasts and epithelial mesenchymal transformation of CRC cells via the ERK signaling pathway, thereby promoting invasion and metastasis in CRC.

## Introduction

Colorectal cancer (CRC) is the second- and third-most common cancer in women and men worldwide 1. Globally, in 2018, new CRC diagnoses included a total of 1,849,518 cases, accounting for about 10% of all cancer and new deaths at 5% 2. Treatment strategies developed for primary and metastatic CRC include laparoscopic surgery for primary disease, resection of metastatic disease affecting the liver and lungs, radiotherapy for rectal cancer and some forms of metastatic disease, along with neoadjuvant and palliative chemotherapy 3-5. Despite their widespread application, the case fatality rates and long-term survival rates of CRC have not improved over the last few decades. Thus, it remains necessary to understand the molecular drivers of this disease and on their basis, develop new therapies for CRC.

Cancer associated fibroblasts (CAFs) are a focus area in research concerning cancer invasion and tumor microenvironment (TME), both of which play pivotal roles in tumor progression 6. Increasing evidence shows that cell interactions between cancer cells and stromal cells surrounding the TME play an important role in regulating cancer progression and treatment response 7. CAFs are important elements of the TME, and can interact with cancer cells to promote stemness, epithelial-mesenchymal transition (EMT), metastasis, and chemotherapy resistance of CRC cells 8. With the recognition of this association, CAFs are recognized as a potential target for anti-cancer treatment. However, the CAFs-derived molecular processes that regulate CRC metastasis and chemoresistance have not been fully characterized. Previous evidence has shown that ANGPTL4 promotes EMT of CRC tumor cells by affecting the activation of CAFs 9. Another study reported that CAFs regulated endothelial protein lipoma-preferred partner through calcium-dependent signaling pathways involving ERK, which in turn affected molecular mechanism pertaining to CAFs behavior in the TME 10. Previous research results also support the idea that ANGPTL4 can inhibit the metastasis of CRC and improve the TME 11. Notably, CAFs in patients with primary tumors, regional metastases, or distant metastases have low expression of ANGPTL4 12. In this study, we aimed to analyze clinical samples of CRC for the expression level of ANGPTL4 gene in the tumor tissue of CRC patients, detect the DNA methylation level of ANGPTL4 promoter in CRC cell lines, and to further study cancer-related fibroblasts during TME changes.

## Materials and methods

### Bioinformatic analysis

The Gene Expression Omnibus (GEO) database (https://www.ncbi.nlm.nih.gov/geo/) was searched and a CRC-related gene expression microarray, GSE10950 was obtained, which included 24 normal control samples and 24 tumor samples. Differential analysis was conducted using the R package "limma" and p-values were corrected using the false discovery rate method, with |logFC| > 1 and FDR < 0.05 as the differential gene screening criteria. ANGPTL4 expression in colon and rectal cancers in The Cancer Genome Atlas (TCGA) and GTEx was searched using the Gene Expression Profiling Interactive Analysis (GEPIA) database (http://gepia2.cancer-pku.cn/#analysis). Using the UALCAN database (http://ualcan.path.uab.edu/cgi-bin/ualcan-res.pl), methylation of the ANGPTL4 promoter region was determined in the TCGA database. The GeneMANIA database (http://genemania.org/) was utilized to obtain ANGPTL4-related genes and gene association network maps were obtained. Thereafter, using the KOBAS3.0 database, KEGG pathway enrichment analysis was performed for the ANGPTL4-related genes.

### Clinical Samples

Informed consent was obtained from all study participants. The study protocol was approved by the ethics committee of the Beijing Chaoyang Hospital. CRC tissues and para-carcinoma tissues were obtained from 67 CRC patients after surgery during Dec 2011 to June 2013. The clinical information of patients is collated in Table 1. All included patients had not received anti-tumor therapy before surgery. Colorectal resection was performed on the basis of preoperative imaging, and postoperative pathological sections were confirmed as CRC. Patients in the control group were age-matched healthy individuals who needed pathological diagnosis and routine colonoscopy, were tested and specimens were obtained through colonoscopy. Meanwhile, normal colon tissue specimens were obtained. The basic information of patients was collected in the medical record room. Follow-up was performed for all patients and started from the end of surgery to the end of June 2018, ranging from 4 to 60 months. The relationship between the expression levels of ANGPTL4 and overall survival (OS) was analyzed by Kaplan-Meier survival analysis method.

### Immunohistochemistry (IHC)

Paraffin sections of tissue specimens were dewaxed with xylene and hydrated with ethanol gradient in turn followed by water-based washing. The inactivation of endogenous peroxides was achieved by 20 min dark treatment with 3% hydrogen peroxide and methanol solution. After washing with distilled water and 0.1M PBS, the sections were treated with heated antigen retrieval solution followed by natural cooling. Then the tissue sections were blocked with goat serum (C-0005, Haoran Biotech) for 20 min and stained with ANGPTL4 primary antibody (ab196746, Abcam) overnight at 4°C. The redundant primary antibody was removed by three 0.1M PBS washes, and the sections were consecutively stained with secondary antibody IgG (ab6785, abcam) and HRP labeled streptomycin ovoalbumin working solution (0343-10000U, Yimo Biotech) for 20 min at 37°C, with the target signal visualized by DBA (ST033, Weijia Biotech). After hematoxylin staining, dehydration and xylene transparency, the sections were mounted with neutral resin and captured with the microscope camera. Five randomly selected views were captured from each section and 100 cells were counted in each view. Semi-quantitative analysis was applied to score the percentage of positive cells and staining intensity under the microscope. To determine the number of positive cells: 5 high-power fields in each section were observed, and the percentage of positive cells was counted, with 0 for the percentage of positive cells < 5%, 1 for 5% to 25%, 2 for 26% to 50%, 3 for 51% to 75%, and 4 for 76% to 100%. (2) To determine positive staining intensity: 0 indicated colorless, 1 indicated yellowish, 2 indicated brownish-yellow, and 3 indicated tan. (3) The positive grade obtained by multiplying the scores of the two: 0 indicated negative (-), 1-4 indicated weakly positive (+), 5-8 indicated positive (+ +), and 9-12 indicated strongly positive (+ + +).

### Cell Culture and Transfection

The normal colonic epithelial cell line NCM460 and the CRC cell lines including SW480, HCT116, DLD1, SW1116, Caco-2, SW620 and HT-29 were purchased from ATCC and cultured with DMEM culture medium (Gibco, USA) containing 10% fetal bovine serum (FBS, Gibco, USA), 10 μg/mL streptomycin and 100 U/mL penicillin at 37°C in a 5% CO_2_ incubator (Thermo Fisher, USA). For cell transfection, cells in the logarithmic phase were detached with trypsin and seeded in six-well plates at a density of 1×10^5^ cells per well. After 24 h culture, when the cell confluence reached about 75%, the transfection was performed using a FuGENE6 transfection kit (Promega, USA) following the standard instructions provided. Following another 48 h of culture, the transfected cells were harvested and the ANGPTL4 expression was quantified using RT-qPCR. Activation of the ERK pathway was performed by treatment with 10 µM of Ceramide C6 (Santa Cruz Biotechnology, Santa Cruz, CA, USA) 13. The cells were divided into shCtr group (transfected with silencing negative control plasmid), shANGPTL4 group (transfected with shANGPTL4 plasmid), control group (transfected with negative control overexpression plasmid), ANGPTL4 group (transfected with oe-ANGPTL4 plasmid), and ANGPTL4 + Ceramide C6 group (transfected with oe-ANGPTL4 plasmid + Ceramide C6).

### RNA extraction and reverse transcription quantitative polymerase chain reaction (RT-qPCR)

Total RNA from tissues or cultured cells was extracted using the Trizol (15596026, Invitrogen) extraction method and then reverse transcribed into cDNA using a PrimeScript RT reagent Kit (RR047A, Takara) according to the manufacturer's instructions. The Fast SYBR Green Mix (Applied biosystems) and its corresponding reaction system were applied to determine the level of target gene expression using an ABI PRISM 7300 RT-qPCR system (Applied biosystems), with previously reported reaction conditions 14, 15. The relative expression of ANGPTL4 to reference gene GAPDH was determined using the 2^-ΔΔC^ method. The primers sequences are shown in Table S1.

### Bisulfite Pyrosequencing

The DNA isolation and subsequent bisulfite conversion were carried out as previously described by using EZ DNA Methylation-Direct^TM^ kit (Zymo Research, Irvine) 16. In brief, according to the kit instructions, the samples were individually washed with PBS and detached using 20 μL proteinase K digestive buffer at 50°C for 2 h. The detached samples were then transferred to PCR tubes containing 130 μL CT convert reagent for 8 min DNA denaturation at 98°C followed by 3.5 h bisulfite conversion at 64°C in a thermal cycler (MJ Research). The bisulfited DNA was then desalted, purified, and eluted with 15 μL elution buffer. Afterwards, 2 μL of the bisulfited DNA was used for bisulfite pyrosequencing polymerase chain reaction (BS-PCR). Here, the reaction mix included 25 μL Zymo Taq premix, 2 μL bisulfited DNA, 21 μL dH_2_O and 1 μL forward and reverse primers. The reaction was programed with 95°C pre-denaturation for 4 min, 40 cycles of 95°C denaturation for 30 sec, 46°C anneling for 30 sec and 72°C extension for 20 sec, and final 72°C extension for 7 min. The PCR product was subjected to agarose gel isolation and purification with TIANgel Midi Purification Kit (Tiangen). The purified DNA segments were cloned into the pMD18-T plasmids (Takara) which were sequenced after PCR verification. Three replicate experiments were performed for each sample, and three to four clones were selected for sequencing in every amplification experiment. The bisulfite pyrosequencing data and C-T conversion rate were analyzed using BIQ software, and the data with C-T conversion rate lower than 95% was excluded. DNA methylation status was analyzed by calculating the percentage of methylated CpG in the total CpG.

### Methylation Specific PCR

Genomic DNA of cultured cell lines was extracted using DNeasy Blood & Tissue Kit (Qiagen GmbH) and bisulfite conversion was performed using EZ DNA Methylation-Gold^TM^ Kit (ZYMO Research) according to the manufacturer's instructions. Next, PCR amplification of the bisulfited DNA was performed with ANGPTL4-methylated or ANGPTL4-nonmethylated specific primers to amplify specific ANGPTL4 promoter regions. The PCR products were separated and visualized by 1.5% agarose gel electrophoresis with commercial methylated DNA (Merck Millipore, Billerica, MA, USA) as positive control and distilled water as negative control. The primer sequence for methylated ANGPTL4 was: R: 5'-TTTAGGTTGGAGCGTAATGGC-3', F: 5'-CAATAACGAAAAAAACGCACG-3'; unmethylated ANGPTL4: R: 5'-CACAATAACACCATCTCAACA-3', F: 5'-TAGTTGGGTTTGGTAGTGTGT-3'.

### 5-Aza-dC Treatment

CRC cells were cultured for 72 h in medium containing 10 µM demethylating agent 5-Aza-DC (Sigma-Aldrich; MerckKGaA, Darmstadt, Germany). The medium was replaced every 24 h with new medium containing 5-Aza-DC, and untreated cells were used as controls. Total RNA was purified and RT-qPCR was performed to quantify ANGPTL4 expression.

### Western blot

Cultured cells were detached and harvested with trypsin, washed with PBS, and lysed with enhanced RIPA lysis buffer (Boster Bio Tech, Wuhan, China) containing 1% proteinase inhibitor cocktail. Whole protein solution was then obtained by cryogenic high-speed centrifugation for the homogenized lysates and quantified using BCA protein assay kit (Boster Bio Tech, Wuhan, China) according to the manufacturer's instructions. A total of 30 μg of whole cell protein was used to perform 10% SDS-PAGE isolation and then electroblotted to PVDF membranes. Thereafter, the membranes were blocked with 5% BSA for 2 h at room temperature and then incubated with the diluted primary antibody overnight at 4°C. The diluted primary antibodies used here were rabbit-anti E-Cadherin (1 : 10000, ab40772, Abcam), rabbit-anti Vimentin (1 : 1000, ab92547, Abcam), rabbit-anti ZO-1 (1 : 10000, ab96587, Abcam), rabbit-anti N-Cadherin (1 : 1000, ab18203, Abcam), rat-anti ERK1 (1 : 1000, ab119357, Abcam), rabbit-anti AKT (1 : 1000, ab38449, phospho T308, Abcam), and rabbit-anti GAPDH (1 : 10000, ab181602, Abcam). After three washes with PBST, the blots were incubated with specific HRP conjugated secondary antibodies to goat anti-rabbit (ab205718, 1:2000, Abcam, Cambridge, UK) or goat anti-rat (ab205719, 1:2000, Abcam, Cambridge, UK) for 1 h at room temperature, and developed and fixed with 1 min ECL (EMD Millipore, USA) working solution incubation and 5~10 min X-ray film exposure in dark conditions. The blot band density was analyzed using Image J software where GAPDH was used as internal reference. Each experiment was performed thrice independently.

### Cell counting kit-8 (CCK-8) Assay

Cell viability was tested using CCK-8 kit (Dojindo Laboratories, Kumamoto, Japan). Briefly, a total of 1 × 10^3^ cells were seeded in each well of a 96-well plate. After incubation for 24, 48, and 72 h, CCK-8 solution was added to each well. Finally, the absorbance was detected using a microplate reader (MolecularDevices, Sunnyvale, CA, USA) at 450 nm in each well.

### Scratch Assay

Parallel lines were drawn at 0.5~1 cm intervals on the bottom of the 6-well plate, with at least five lines in each well. The cultured or treated cells were detached and seeded into the labeled 6-well plate at the density of 5×10^5^ cells/well. After overnight culture, a sterile 10 µL pipette tip was used to scratch a wound perpendicular to the horizontal lines on the back of plate. Images were captured, the scar distance was measured and recorded under an optical microscope camera after 0 h and 24 h culture and cell migration was determined for each group.

### Transwell Assay

The invasion competence of CRC cells was assayed by evaluating their ability to invade through a synthetic basement membrane, growth factor-reduced Matrigel matrix (356230, BD Biosciences, Frankin, NJ, USA). The experiment was performed as previously described 17. Briefly, the transwell inserts (3422, Costar, Corning, NY, USA) were first coated with growth factor reduced Matrigel and placed in a 37°C incubator for 30 min for Matrigel polymerization, and the basement membrane was ordinarily humidified with culture medium before use. Thereafter, cells subjected to 12 h starvation were harvested, resuspended in serum free culture medium at a concentration of 1 × 10^5^/ml, and 100 µl cell suspension was seeded in the insert separately. After 24 h incubation at 37°C, the non-invaded cells on the upper surface of the membrane were removed using a cotton swab while the remaining cells were fixed with 100% methanol and stained with 1% toluidine blue. Stained cells on the bottom membrane were captured using an inverted light microscope (CarlZeiss, Germany), five views in each capture were selected randomly, and the number of stained cells was counted. All experiments were carried out in triplicate.

### Immunofluorescence

Cells were firstly seeded in the culture plate lined with slides. After conditional treatment, the slides with treated cells were fixed in 4% PFA for 15 min and permeated in 0.5% Triton X-100/PBS for 20 min (the procedure should be skipped upon detecting antigens expressed on cell membrane). After three PBS washes, the fixed cells were blocked using 3% bovine serum albumin (BSA) for 30 min at room temperature and then incubated with diluted primary antibody (Anti-alpha Smooth Muscle Actin (ab5694, Abcam); Vimentin (1 : 250, ab92547, Abcam)) overnight at 4°C. Binding of the primary antibody was visualized by goat anti-human antibody conjugated with TRITC secondary antibody. The cell nuclei were stained with DAPI. After mounting, the immunofluorescence results were captured and analyzed on a Leica TCSNT confocal system (Leica, Wetzlar, Germany).

### Isolation of Cancer Associated Fibroblasts

Fresh collected CRC tissues were immediately washed with cold sterile PBS to remove blood clots, sheared into pieces, and enzymatically detached with 2 mg/ml type I collagenase (Gibco, USA) for 30 min at 37°C. The mixture was then transferred into a 50 ml sterile centrifuge tube, vibrated and centrifuged at 300 rpm for 3 min. The cell suspension was discarded, and the tissue deposit was cultured in an incubator and then detached with collagenase solution. This was repeated thrice, and loose tumor tissues were obtained, further detached with 0.25% trypsin for 10 min at room temperature, and then inactivated by adding appropriate volume of FBS. The tumor cell pellets were obtained by centrifuging the above digestion mixture and resuspended in DMEM containing 10% FBS. Tumor associated fibroblasts were obtained following several times passages, and the cell purity was evaluated by flow cytometry.

### Tumorigenesis in nude mice

Seventy-two healthy male SCID nude mice (6 - 8 weeks old) were obtained from Beijing Vital River Laboratory Animal Technology Co., Ltd. and housed in specific-pathogen-free facilities with humidity at 60%~65%, temperature at 22~25°C and a 12-h light/dark cycle. Sterile food and water were provided *ad libitum*. After one-week of adaptive feed, the tumorigenesis experiments were performed as previously described, 18 chiefly by tail vein injection of 1 × 10^6^ HCT116 cell lines treated with shCtr, HCT116 cell lines with shANGPTL4, Control HCT116 cell lines, HCT116 cell lines with oe-ANGPTL4, Control HCT116 cell lines + cancer associated fibroblasts (CAF) cell lines (3 : 1), or HCT116 cell lines with oe-ANGPTL4 + CAF cell lines (3 : 1) into the nude mice, respectively. During tumor formation, cell metastasis in the lung tissues was monitored by bioluminescence imaging. About six weeks later, the mice were sacrificed and their lung tissues were collected for pathological analysis. All animal experiments were carried out in accordance with guidelines approved by the Institutional Animal Care and Use Committee.

### Statistical analysis

All descriptive data in this study were summarized as mean ± standard deviation. Statistical analysis was performed using SPSS 21.0 (IBM, USA) software. Paired t-test was used to analyze the difference between two groups, while the one-factor analysis of variance (ANOVA) and Tukey's post-hoc test were employed to analyze the difference between multiple groups. The survive rate was calculated by Kaplan-Meier plotter and the corresponding single factor analysis was performed by Log-rank test. The differences were considered significant for* p* < 0.05.

## Results

### ANGPTL4 expression was decreased in CRC tissues and cells and was associated with poor prognosis of CRC patients

**Figure 1:**
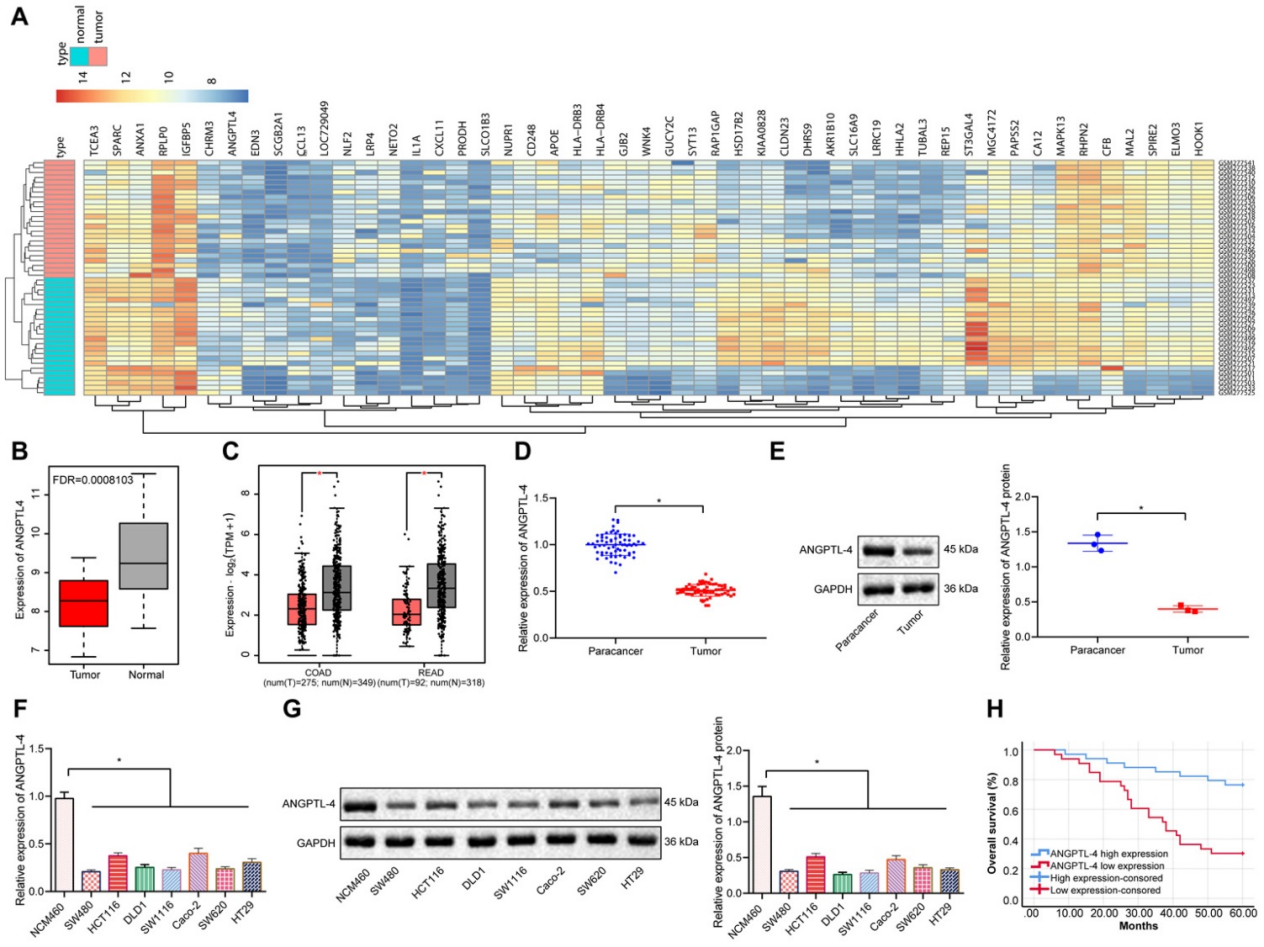
** ANGPTL4 expression was reduced in CRC tissues and cells and was associated with poor prognosis of CRC patients.** (A) Heatmap depicting partially significant differential gene expression in the CRC-associated gene expression microarray dataset GSE10950; x-axis indicates sample number, y-axis indicates gene name, left dendrogram indicates gene expression level cluster, upper dendrogram indicates sample cluster, each small square indicates the expression of a gene in a sample, and upper right histogram indicates color order (n = 24). (B) Differential gene expression analysis of ANGPTL4 in the GSE10950 microarray dataset; red box plots indicate tumor samples and gray box plots indicate normal samples, and the upper left corner is the corrected differential p-value (n = 24). (C) The expression levels of ANGPTL4 in CRC samples included in the TCGA database; the left side represents colon cancer and the right side represents rectal cancer, the red box plot indicates tumor samples, and the gray box plot indicates the normal samples (n = 24, * *p* < 0.05 vs. the tumor adjacent tissue). (D) RT-PCR analysis of mRNA expression of ANGPTL4 in CRC tissues (n = 67, * *p* < 0.05 vs. the para-carcinoma tissues). (E) Western blot analysis of protein levels of ANGPTL4 in CRC tissues, * *p* < 0.05 vs. the tumor adjacent tissue. (F) RT-qPCR determination of ANGPTL-4 mRNA expression levels in CRC cell lines, * *p* < 0.05 vs. the NCM460 cells. (G) Western blot analysis of ANGPTL-4 mRNA expression levels in CRC cell lines, * *p* < 0.05 vs. the NCM460 cells. (H) Survival rate determined by Kaplan-Meier survival curve analysis. All *in vitro* experiments were performed independently at least three times and the representative data were summarized as mean ± standard deviation. In RT-qPCR and western blot experiments, GAPDH served as the internal reference control. Comparisons were made using Student's *t*-test. *p < 0.05.

Differential gene expression analysis using the CRC microarray dataset GSE10950 revealed that ANGPTL4 was significantly under-expressed in CRC (Figure 1A, B). Further analysis of the expression of ANGPTL4 in CRC samples in TCGA similarly revealed that ANGPTL4 was significantly poorly expressed in CRC (Figure 1C). These findings were verified by RT-qPCR and Western blot results in our study, which showed that the expression of ANGPTL4 was significantly decreased in both CRC tissues and CRC cell lines as compared to controls (Figure 1D-G). Using seven CRC cell lines, we noted that the mRNA and protein expression of ANGPTL4 was relatively low in SW480 cells, but relatively high in HCT116 cells. Therefore, we selected SW480 and HCT116 cells for the subsequent experiments. Survival analysis showed that lower expression of ANGPTL4 in CRC tissues was significantly correlated with higher pathological stages, and ANGPTL4 expression was positively correlated with the survival rate of patients (Figure 1H). These results supported the notion that ANGPTL4 was poorly expressed in CRC tissues and cells, and was significantly associated with poor patient prognosis.

### DNA methylation regulated the expression of ANGPTL4 in CRC

**Figure 2:**
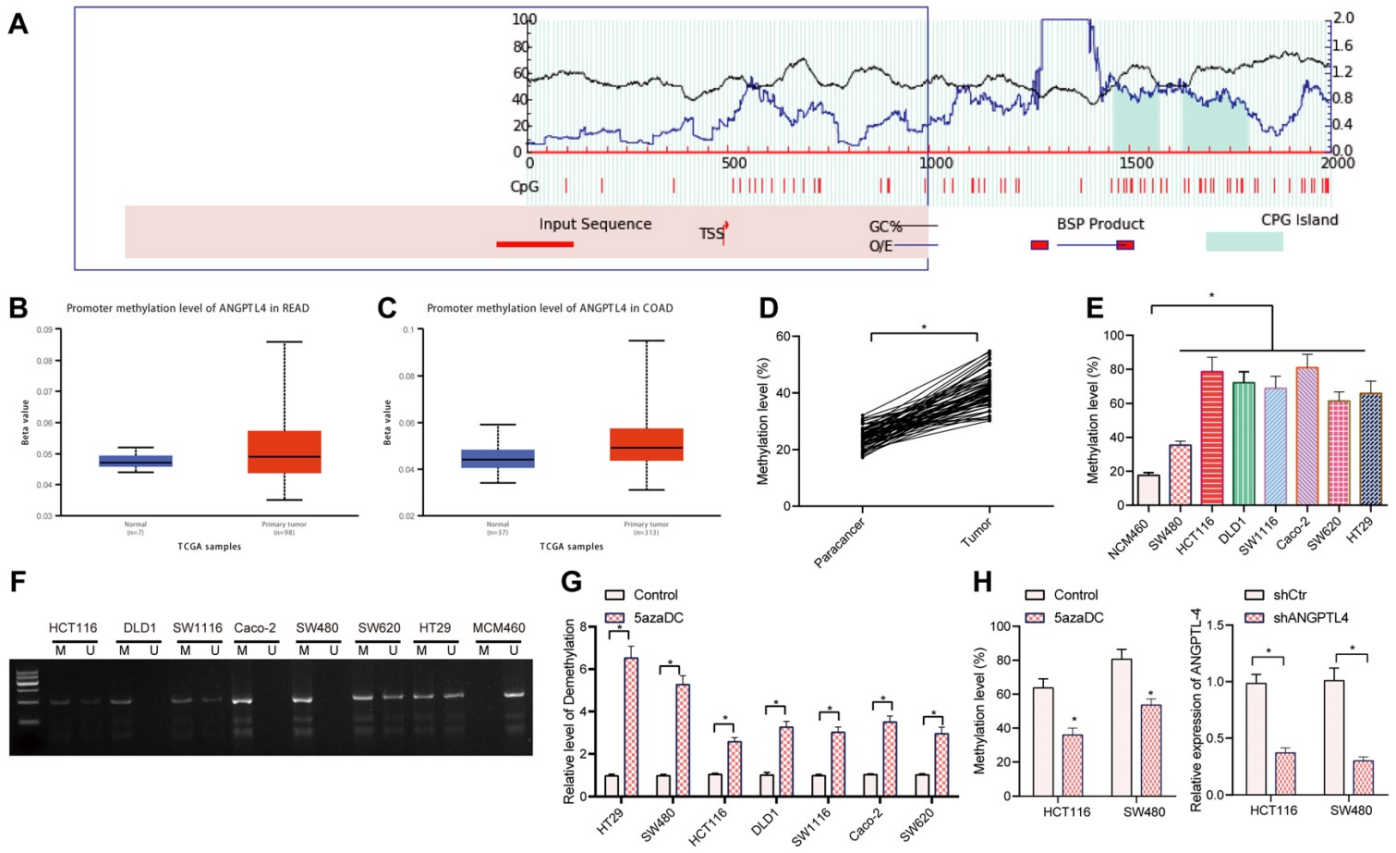
** DNA methylation mediated the expression of ANGPTL4 in CRC.** (A) Methylation islands of genes predicted using MethPrimer. (B) Methylation of the promoter region of ANGPTL4 in rectal cancer samples included in the TCGA database; blue boxplots indicate normal samples and red boxplots indicate tumor samples (n = 24, B: *p* = 1.56280E-04, C: *p* = 3.40250E-12). (C) Methylation of the promoter region of ANGPTL4 in CRC samples included in the TCGA database; blue boxplots indicate normal samples and red boxplots indicate tumor samples (n = 24, B: p = 1.56280E-04, C: *p* = 3.40250E-12). (D) Bisulfite sequencing analysis of methylation levels in CRC clinical samples (n = 67, * *p* < 0.05 vs. para-carcinoma tissues). (E) Bisulfite sequencing was used to determine the methylation level of ANGPTL4 promoter region in CRC cell lines (* *p* < 0.05 vs. NCM460 cells). (F) Methylation-specific PCR was used to determine the methylation status of each CRC cell line. (G) RT-qPCR was used to determine the expression levels of ANGPTL4 in CRC cell lines after 5-aza-DC treatment (* *p* < 0.05 vs. the control group). (H) Bisulfite sequencing was used to detect the methylation level of ANGPTL4 promoter region in SW480 and HCT116 cells after 5-aza-DC treatment (* p < 0.05 vs. the control group).

It has been documented that DNA methylation is involved in the regulation of ANGPTL4 expression 19. Methylation islands of genes predicted by using MethPrimer revealed the presence of CpG islands in the promoter region of ANGPTL4 (Figure 2A). Further search for ANGPTL4 promoter methylation in CRC using TCGA revealed that ANGPTL4 promoter was hypermethylated in CRC tissues (Figure 2B, C). Therefore, we speculated that DNA methylation is involved in regulating the expression of ANGPTL4 in CRC. Bisulfite sequencing results showed that ANGPTL4 promoter methylation was significantly increased in CRC tissues (Figure 2D). At the same time, we also measured ANGPTL4 expression levels in CRC cell lines, and found that 6 (86%) of 7 CRC cell lines had higher methylation levels of ANGPTL4 (Figure 2E, F). Next, the CRC cell lines were treated with 5-aza-DC. RT-qPCR results showed that 5-aza-DC treatment significantly up-regulated ANGPTL4 expression in CRC cells (Figure 2G). Bisulfite sequencing showed that 5-aza-DC treatment significantly decreased methylation in CRC cells (Figure 2H). Taken together, DNA hypermethylation was found involved in the down-regulation of ANGPTL4 in CRC.

### Silencing of ANGPTL promoted proliferation, migration, invasion, and EMT of CRC cells

**Figure 3:**
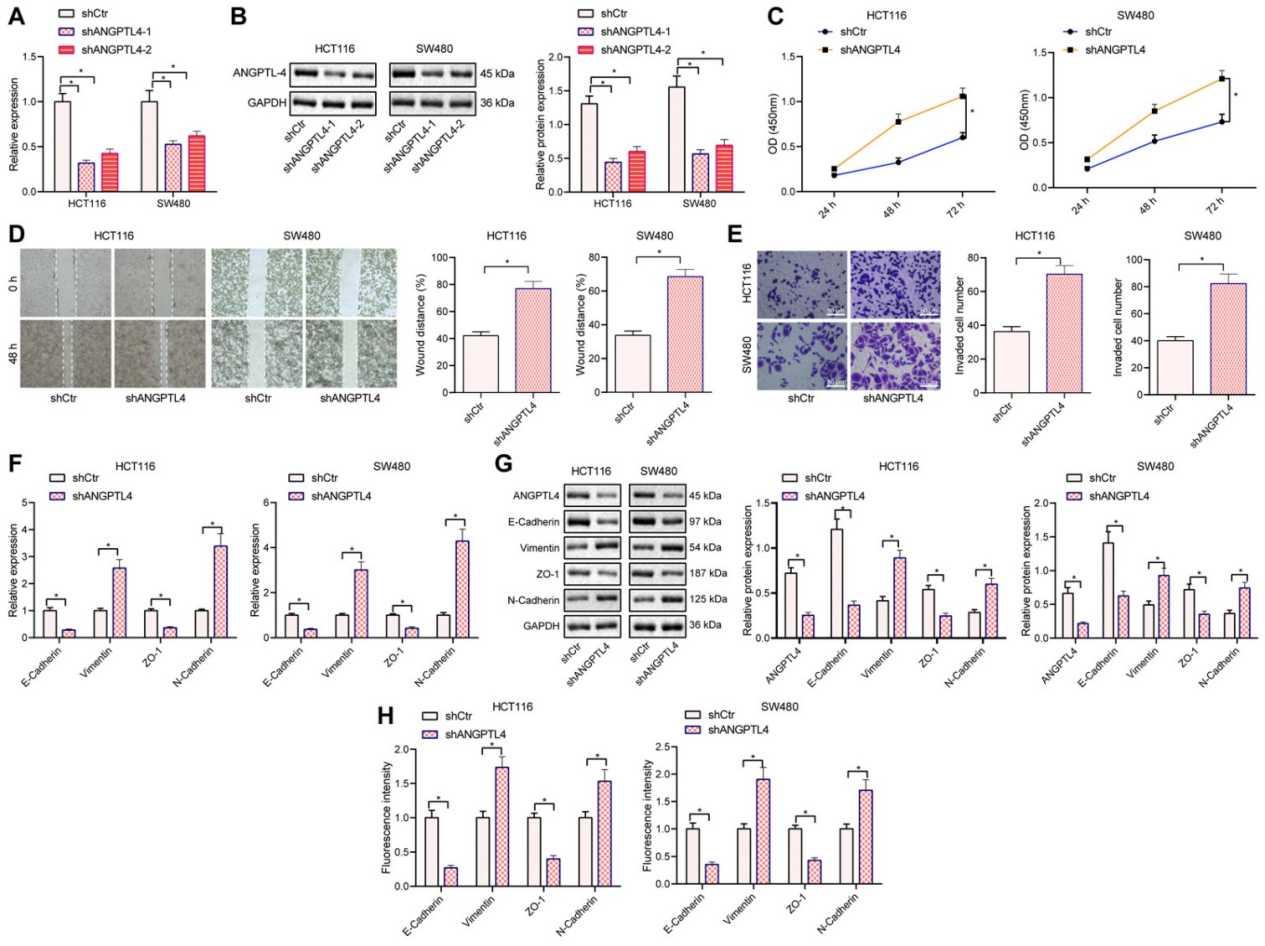
** Knockdown of ANGPTL4 promoted proliferation, migration, invasion, and EMT of CRC cells.** (A) RT-qPCR to determine the silencing efficiency of ANGPTL4 in HCT116 and SW480 cells. (B) Western blot was used to detect the silencing efficiency of ANGPTL4 in HCT116 and SW480 cells. (C) CCK8 assay to quantify the proliferation of HCT116 and SW480 cells. (D) Scratch assay was used to determine the migration ability of HCT116 and SW480 cells. (E) Transwell assay was used to determine the invasion ability of HCT116 and SW480 cells (200×). (F) Western blot analysis was used to detect the expression levels of EMT-related markers in CRC cells after ANGPTL4 knockdown. (G) RT-qPCR was used to quantify the expression levels of EMT-related markers in CRC cells after ANGPTL-4 knockdown. (H) Immunofluorescence was used to determine the expression levels of EMT-related markers in CRC cells after ANGPTL-4 knockdown, E-Cadherin, Vinmentin, ZO-1, and N-Cadherin were all localized in the cell membrane, and electron microscopy was used to observe the morphology of CRC cells with ANGPTL-4 knockdown. * *p* < 0.05.

To investigate the effect of ANGPTL4 on the migration and invasion of CRC cells, we silenced ANGPTL4 in CRC cell lines HCT116 and SW480. RT-qPCR and Western blot results displayed that shANGPTL4 treatment could reduce the expression of ANGPTL4 in the two CRC cell lines, and the silencing efficiency of shANGPTL4-1 was highest, so the shANGPTL4-1 sequence was selected for subsequent experiments (Figure 3A, B). The results of CCK8 assay, scratch test, and transwell assays showed that silencing ANGPTL4 significantly promoted the proliferation, migration, and invasion of CRC cells, respectively (Figure 3C-E). EMT is essential for tumor metastasis and invasion 20 and given our results showing ANGPTL4 could regulate the migration and invasion of CRC cells, we asked whether ANGPTL4 was involved in the EMT process of CRC cells. Western blot and RT-qPCR results revealed that the expression of epithelial markers E-Cadherin and ZO-1 was significantly down-regulated, and the expression of mesenchymal cell markers vimentin and N-Cadherin was significantly up-regulated in CRC cells after shNAGPTL4 treatment (Figure 3F, G). Immunofluorescence results also showed that upon shANGPTL4 treatment, the CRC cells underwent EMT (Figure 3H).. In sum, these data indicated that silencing ANGPTL4 could promote the proliferation, migration, invasion, and EMT of CRC cells.

### Silencing ANGPTL4 enhanced migration, invasion, and EMT of CRC cells by activating the ERK signaling pathway

**Figure 4:**
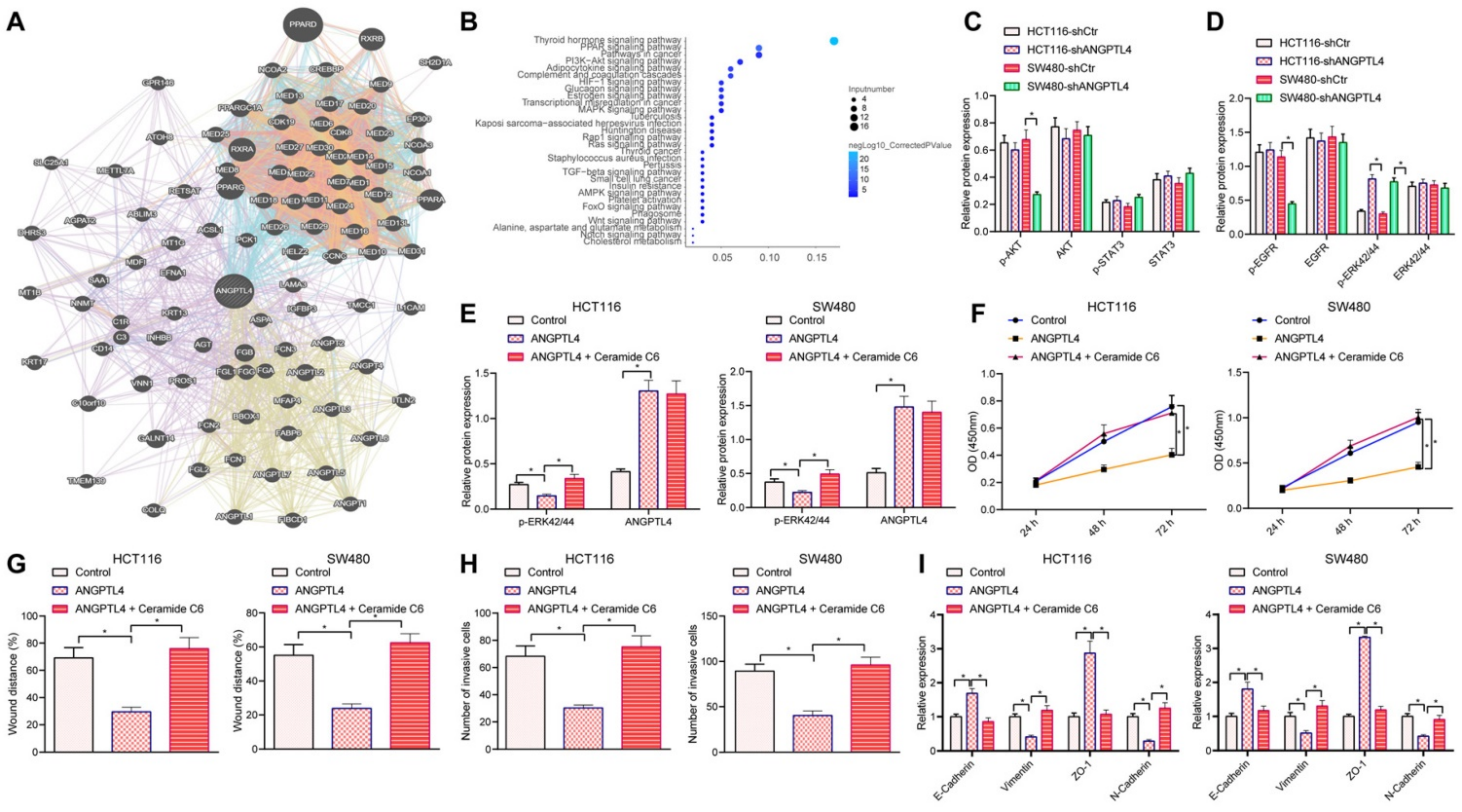
** ANGPTL4 knockdown regulated the migration, invasion, and EMT of CRC cells by activating ERK signaling pathway.** (A) ANGPTL4-related genes were predicted *in-silico*, and each circle indicates a gene and the line between circles indicates an association between genes. (B) KEGG pathway enrichment analysis of ANGPTL4-related genes, wherein the x-axis indicates Gene Ratio, y-axis indicates KEGG items, the circle size indicates the number of genes enriched in the items, the circle color indicates the enrichment p value, and the right histogram is the color scale. (C) Western blot analyses to detect the activation status of p-AKT/AKT and p-STAT3/STAT3 after ANGPTL4 knockdown. (D) Western blot analysis to detect the activation status of p-EGFR/EGFR and p-ERK42/44/ERK42/44 after ANGPTL4 knockdown. (E) Western blot analysis to detect the expression level of p-ERK42/44 in HCT116 cells and SW480 cells after ANGPTL4 or Ceramide C6 treatment. (F) CCK8 assay to quantify cell proliferation. (G) Wound healing assay to determine the cell migration ability. (H) Transwell assay to determine cell invasion ability. (I) RT-qPCR to determine the mRNA expression levels of EMT-related proteins in CRC cells. (J) Western blot assay to determine the expression level of EMT-related proteins. * *p* < 0.05.

In order to explore the molecular mechanisms by which ANGPTL4 could regulate the invasive ability of CRC cells, we searched for ANGPTL4-related genes and obtained 100 such genes (Figure 4A). These related genes were subjected to KEGG pathway enrichment analysis and found to be mainly enriched in signaling pathways including PI3K-AKT (Figure 4B). It has been reported that ERK, PI3K/AKT, WNT, and other signaling pathways are associated with deterioration in CRC and are also noted as potential regulators of the EMT process 21-23. Western blot results revealed that the levels of p-ERK42/44 were significantly up-regulated in CRC cells upon silencing ANGPTL4 (Figure 4C, D). Further, we speculated that silencing ANGPTL4 promoted CRC cell migration, invasion, and EMT by activating ERK. Western blot results showed that the expression of ANGPTL4 was significantly up-regulated and the expression of p-ERK42/44 was significantly down-regulated in CRC cells overexpressing ANGPTL4; however, this decrease in levels of p-ERK42/44 in CRC cells was significantly reversed after co-treatment with the specific ERK1/2 activators Ceramide C6 and ANGPTL4 (Figure 4E). The results of CCK8, wound healing, and Transwell assays indicated that overexpression of ANGPTL4 inhibited the proliferation, migration, and invasion of CRC cells, while co-treatment with Ceramide C6 and ANGPTL4 counteracted this inhibitory effect (Figure 4F-H). RT-qPCR and Western blot results demonstrated that overexpression of ANGPTL4 up-regulated the expression of epithelial markers E-Cadherin and ZO-1 and down-regulated the expression of mesenchymal cell markers vimentin and N-Cadherin, while co-treatment with CeramideC6 and ANGPTL4 reversed these results (Figure 4I-J). Altogether, the results demonstrated that silencing ANGPTL4 induced CRC cell migration, invasion, and EMT by activating the ERK signaling pathway.

### Stromal fibroblasts were activated in the tumor microenvironment of colorectal patients

**Figure 5:**
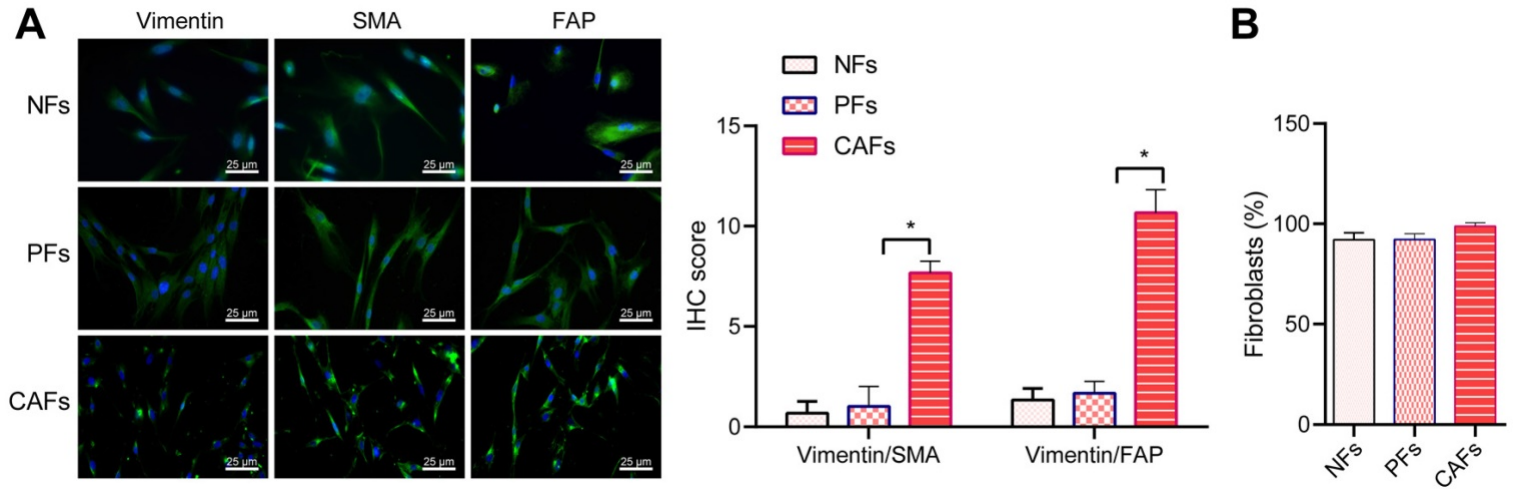
** Stromal fibroblasts are activated in the tumor microenvironment of CRC patients.** (A) Immunofluorescence staining for Vimentin (red), α-SMA (green) and FAP (green) in the isolated primary fibroblasts (400×). NF, normal fibroblasts; PF, pericancerous fibroblasts; CAF, cancer associated fibroblasts. Nuclei (blue) were labelled by DAPI. (B) Flow cytometry analysis of the expression of Vimentin in the three types of isolated primary fibroblasts. All experiments were performed independently at least three times

Tumor cells derived TGF-beta and SDF-1 is reported to activate fibroblasts and further promote malignant transformation and metastasis 24 and cancer associated fibroblasts are reportedly continuously in an active state and lose the ability to apoptosis 25. Thus, we performed pathological staining to study the activation of fibroblasts in CRC tissues. Immunofluorescence staining showed α-SMA positive cells and FAP positive cancer associated fibroblasts in the CRC area and the peri-cancerous area (Figure 5A), suggesting that stromal fibroblasts in colorectal tumor microenvironment were activated. In order to confirm such change in cancer associated fibroblasts in CRC, we successfully isolated fibroblasts from carcinoma tissues, para-carcinoma tissue, and the normal tissues obtained, which were termed cancer associated fibroblasts (CAF), pericancerous fibroblasts (PF) and normal fibroblasts (NF), respectively. All three types of fibroblasts showed the typical characteristics of fibroblasts featuring long and spindle shape and were positive for fibroblasts cell-markers including Vimentin (Figure 5A). As compared with peri-cancerous fibroblasts and normal fibroblasts, the cancer associated fibroblasts showed increased expression of alpha SMA and FAP (Figure 5A). The flow cytometry analysis showed that the expression of Vimentin was also elevated in cancer associated fibroblasts (> 99%) (Figure 5B). These results confirmed the activation of stromal fibroblasts in the tumor microenvironment of CRC.

### Silencing ANGPTL4 activated fibroblast signaling in the tumor microenvironment and ultimately promoted cancer metastasis

**Figure 6:**
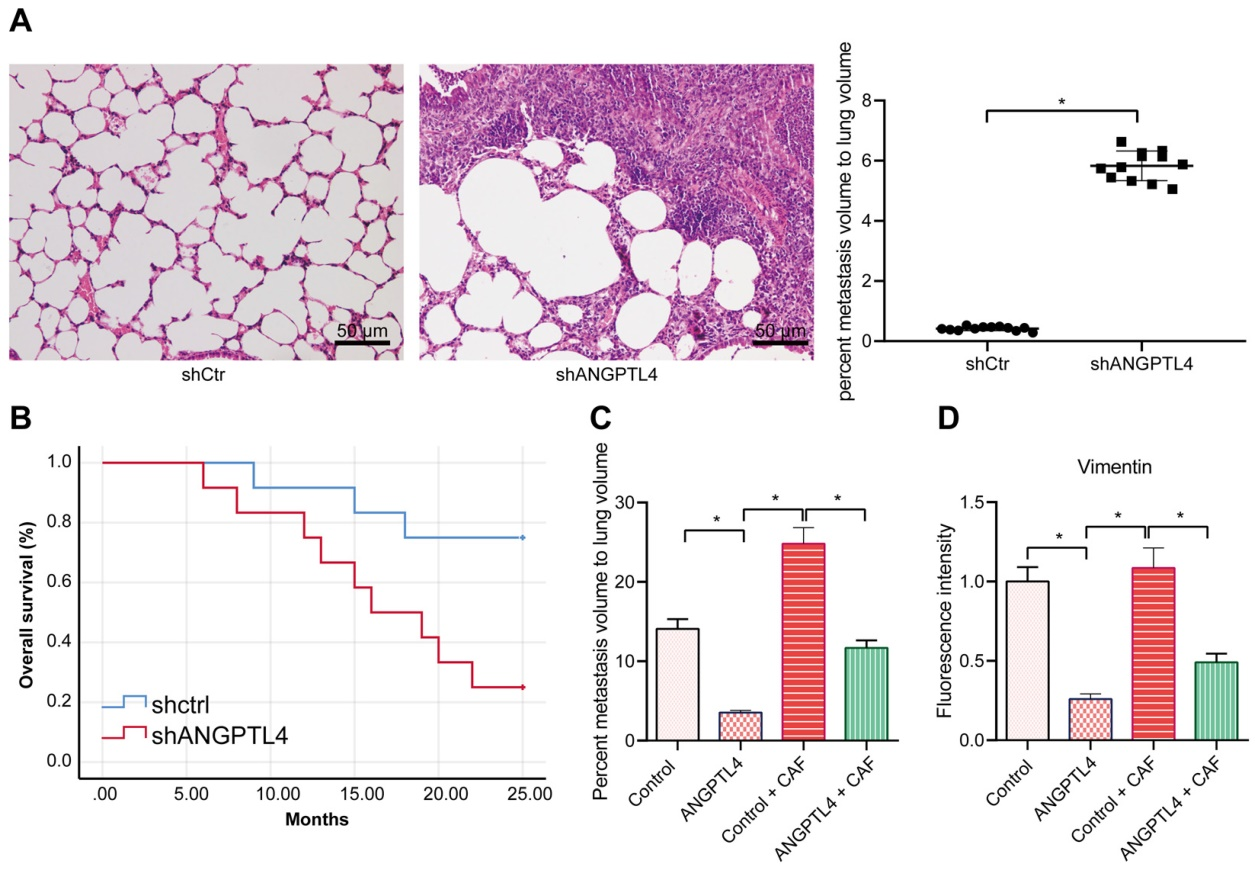
** Silencing ANGPTL4 activates fibroblast signaling in the tumor microenvironment and ultimately facilitates CRC metastasis.** (A) Hematoxylin-eosin staining analysis of lung tissues in tumor mice model (200×). (B) Survival analysis of the tumor nude mice. (C) Hematoxylin eosin staining of Vimentin (green) and α-SMA (red) in the lung tissues of tumor mice. (D) Immunofluorescence analysis of α-SMA and vimentin expression in tissues of CRC mouse models. All experiments were performed independently at least three times and the representative data were summarized as mean ± SEM. Two-group differences were analyzed using Student's *t*-test and One-way analysis of variance (ANOVA) was used for multiple group comparisons. *p < 0.05.

To assess whether silencing ANGPTL4 affected the promotion of cancer metastasis by activating fibroblast signaling in the tumor microenvironment, we constructed nude mice models. The results of H&E staining showed that the level of lung metastases was increased in the HCT116 cell line after ANGPTL4 knockdown (Figure 6A). Survival analysis suggested that the prognosis of mice with ANGPTL4 knockdown was poor (Figure 6B). Further examination showed that the mice injected with HCT116 cell line overexpressing ANGPTL4 generated fewer lung metastases, whereas the mice injected with HCT116 cell line overexpressing ANGPTL4 and CAF had more metastases than the mice injected with HCT116 cell line alone with oe-ANGPTL4. There was no significant difference between the mice injected with Control HCT116 cell line alone and the mice injected with Control CAF (Figure 6C). Immunofluorescence results revealed that the expression of vimentin was significantly decreased in the mice injected with HCT116 cell line with oe-ANGPTL4 than in the mice injected with Control HCT116 cell line, while the expression of vimentin was increased in the mice injected with oe-ANGPTL4 + CAF, as compared with the mice injected with oe-ANGPTL4 alone (Figure 6D). Together, these findings depicted that silencing ANGPTL4 in tumor cells ultimately promoted cancer metastasis by activating fibroblast signaling in the tumor microenvironment.

## Discussion

CRC is one of the most aggressive known tumors occurring in the human digestive system, and most CRC patients show poor prognosis because of metastasis and recurrence 26 as metastatic outgrowth is the predominant cause of lethal outcome in human cancers including CRC 27. This study revealed that DNA promoter methylation silenced the ANGPTL4 gene to induce the activation of CAFs in the tumor microenvironment and formed a positive feedback loop with the EMT of CRC cells, ultimately promoting the invasion and metastasis of CRC.

ANGPTL4 has been earlier found transcriptionally repressed by DNA methylation in urothelial carcinoma cell lines and tumor samples 28. Here, in a similar finding we showed that the expression of ANGPTL4 was decreased in CRC tissues and cell lines due to the hyper-methylation of ANGPTL4 promoter, which was significantly correlated with worse prognosis. In another corroborating finding, a previous study reported the mean preoperative plasma ANGPTL4 level in CRC patients (247.2 ± 230.7 ng/mL) was much lower as compared to individuals with benign colorectal disease (330.8 ± 239.0 ng/mL, *p* = 0.01) 29. Furthermore, a stable ANGPTL4-transfected human liver cancer cell line showed a lower proliferation rate and decreased mean volume and weight of tumors 30.

Tumor cells and surrounding stromal cells, including CAFs, macrophages and immune cells, mutually communicate with each other, resulting in tumor progression and treatment resistance 31, 32. In recent years, accruing evidence suggests the crosstalk between malignant cells and CAFs actively promotes tumor growth and metastatic spread, but therapeutic strategies targeting tumor stroma are not currently utilized in clinical practice 33. In this study, we found that ANGPTL4 knockdown resulted in activated CAFs in the tumor microenvironment. Until now, the impact of ANGPTL4 on CAFs was not investigated, and therefore, additional studies are warranted to delineate their links.

Substantial evidence has shown that the EMT is essential for tumor metastasis and invasiveness 34. In view of the fact that ANGPTL4 could regulate the migration and invasion ability of CRC cells, we asked whether ANGPTL4 was involved in the EMT process of CRC, using HCT116 and SW480 cells as models. Induction of molecules involved in the regulation of EMT was earlier shown as dependent on ANGPTL4 expression in head and neck squamous cell carcinoma cells 35. PI3K/AKT, WNT and other signaling pathways are related to the deterioration of CRC and potential regulators of EMT process and we examined the protein levels of ERK, AKT, and WNT with the corresponding activated proteins to show that upon silencing ANGPTL4, the levels of p-ERK42/44 significantly increased in both cell lines. Consistently, the increase in the ANGPTL4 expression was found dependent on the JNK-MAPK signaling pathway in osteoblastic MC3T3-E1 cells 36. Further, ERK has been observed to largely attenuate the anti-apoptotic effect of ANGPTL4 on hypoxia/serum deprivation-induced apoptosis in mesenchymal stem cells 37. The ERK-ZEB1 pathway can activate EMT, advancing the high-metastatic ability of lung cancer cells 38. Recent data has suggested that ARRB1 promotes hepatocellular carcinoma cell invasion and metastasis through p-ERK1/2-mediated EMT, and thus the suppression of p-ERK1/2 may offer potential therapeutic targets for hepatocellular carcinoma therapy 39. Also, a higher dependence on ERK1/2 signaling for cell migration is typically observed in CAFs obtained from cancer tissue 40.

**Figure 7:**
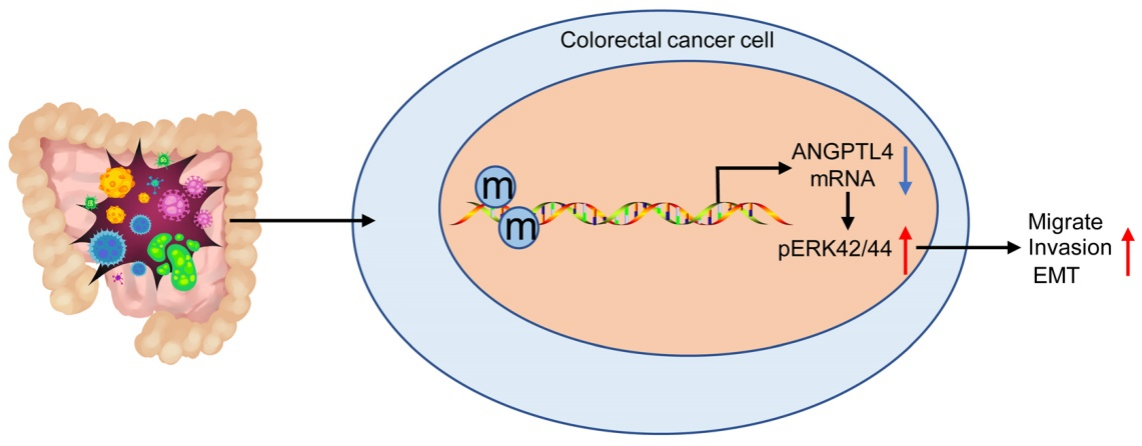
DNA methylation-mediated down-regulation of ANGPTL4 expression promotes tumor metastasis by altering the tumor microenvironment in CRC through activation of the ERK pathway.

In conclusion, our results indicated that DNA methylation-mediated ANGPTL4 gene silencing could induce activated CAFs and facilitate the metastasis of CRC through the ERK/EMT pathway (Figure 7). The newly discovered ANGPTL4/ERK/EMT link in CRC highlights a potential molecular mechanism implicated in CRC development and progression. These data also suggest that ANGPTL4 may serve as an effective therapeutic target for CRC. However, it remains essential to study the precise and comprehensive mechanisms involved in regulating the CRC tumor microenvironment in this context.

## Supplementary Material

Supplementary table.

## Figures and Tables

**Table 1:** Clinical characteristics of 67 CRC patients

Characteristics	No.	Low expression of ANGPTL4 (n)	High expression of ANGPTL4 (n)	*p* value
Age	67			0.549
<50	28	15	13	
>=50	39	18	21	
Gender				0.271
Male	41	18	23	
Female	26	15	11	
Tumor size				0.266
<=5	31	13	18	
>5	36	20	16	
Invasion depth				0.173
M	30	12	18	
S	37	21	16	
Lymph metastasis				0.039
Yes	44	26	18	
No	23	7	16	
Remote metastasis				0.004
Yes	39	25	14	
No	28	8	20	
