# Supplementary material for: DNA methylation mediated down-regulation of ANGPTL4 promotes colorectal cancer metastasis by activating the ERK pathway
Source: J Cancer. 2021 Jul 13;12(18):5473–85. doi: 10.7150/jca.52338 (PMC8364648; doi:10.7150/jca.52338)
Supplement: Supplementary file 1 — Supplementary table. [file jcav12p5473s1.pdf]

Table S1 Primer sequences

Genes	Primer sequences
ANGPTL4	F: 5’-AAAGCAAGGTCTCCCCACAAG-3’<br>R: 5’-GAATTACTGTCCAGCCTCCATC-3’
E-cadherin	F: 5’-GGTAGGTGAATTTTTAGTTAATTAGTGGTA-3’<br>R: 5’-ACCCATAACTAACCAAAAACACCA-3’
ZO-1	F: 5’-TGGACCAGCTAACCAACGACAAAG-3’<br>R: 5’-GGATTGCAGGGTGCTTTCGGCT-3’
vimentin	F: 5’-TCAGAGAGAGGAAGCCTA-3’<br>R: 5’-CTTGACATTGAGAGTTGCCACCCTGCCTGTTGGAAACTTTTCC-3’
N-cadherin	F: 5’-CAACCGTCTTAATCAGGAGTG-3’<br>R: 5’-GTTCGAACGATGGGCAGCCGGTAG-3’
GAPDH	F: 5’-GTGGACCTGACCTGCCGTCT-3’<br>R: 5’-GGAGGAGTGGGTGTCGCTGT-3’
